# Comparative Testing of Two Ligature-Induced Periodontitis Models in Rats: A Clinical, Histological and Biochemical Study

**DOI:** 10.3390/biology11050634

**Published:** 2022-04-21

**Authors:** Darius C. Tomina, Ștefan A. Petruțiu, Cristian M. Dinu, Bogdan Crișan, Vasile S. Cighi, Ioana A. Rațiu

**Affiliations:** 1 Department of Periodontology, Faculty of Dentistry, University of Medicine and Pharmacy Iuliu Hațieganu, 400012 Cluj-Napoca, Romania; tomina.darius@umfcluj.ro; 2 Department of Oral and Cranio-Maxillofacial Surgery, Faculty of Dentistry, University of Medicine and Pharmacy Iuliu Hațieganu, 400012 Cluj-Napoca, Romania; cristian.dinu@umfcluj.ro; 3 Department of Maxillofacial Surgery and Implantology, Faculty of Dentistry, University of Medicine and Pharmacy Iuliu Hațieganu, 400012 Cluj-Napoca, Romania; crisan.bogdan@umfcluj.ro; 4 Faculty of Animal Science and Biotechnologies, University of Agricultural Sciences and Veterinary Medicine, 400372 Cluj-Napoca, Romania; vasile.cighi@usamvcluj.ro; 5 Discipline of Nephrology, Department of Medical Sciences, Faculty of Medicine and Pharmacy, University of Oradea, 410073 Oradea, Romania; ratiu_ioana@yahoo.com

**Keywords:** experimental periodontitis, systemic inflammation, Interleukin-1alpha, tumor necrosis factor-alpha, high sensitive C reactive protein histology

## Abstract

**Simple Summary:**

This study is the first study comparing the same parameters of inflammation in two periodontal disease experimental models proposed by the literature and used in the research. The importance of the method used to induce periodontitis in animals resides in the efficacy of proposed technologies and treatments used in preclinical trials. The inflammatory markers Interleukin-1 alpha(IL-1α), Tumor Necrosis Factor-alpha (TNF-α), and high sensitive C reactive protein (hsCRP), the hematological analyses, and the histological probes showed a similar and reproducible periodontal inflammation for the molar induced periodontitis model. Ligation-induced periodontitis in rats has limitations and will never reproduce all aspects of periodontal disease in humans. The findings of this study with the complex association between clinical, biochemical, and histological aspects of the two experimental models of periodontal pathology induction in rats suggest that a similar periodontal pathology to the one we find in humans is best replicated in rats with the molar induced periodontitis model.

**Abstract:**

Experimental animal models for studying the mechanisms of periodontitis and its links are a better alternative to in vitro studies. The aim of this study is to compare two ligature induced periodontitis models and validate the best one for further use in research. An experimental study was performed on male Wistar rats that were divided into three groups: Test 1 (*n* = 10), incisor ligated, Test 2 (*n* = 10), molar ligated, and Control (*n* = 10). The animals were clinically evaluated at the beginning and at the end of the experiment by recording body weight, gingival bleeding index, tooth mobility score, changes in color, and consistency of gingival tissue. Two blood samples were obtained for each animal at baseline and at the end of the experiment. The hematological parameters Interleukin-1 alpha (IL-1 α), high sensitive C Reactive Protein (hsCRP), and Tumor Necrosis Factor-alpha (TNF-α) were measured. Seven days after the induction of periodontitis, the animals were sacrificed, and samples were prepared for histological evaluation. The results of this research demonstrated that the association between clinical, histological, and biochemical parameters initiate a periodontal pathology in the molar induced model in rats while the incisor experimental model initiates only a moderate and incomplete periodontal inflammation, mainly due to mechanical irritation.

## 1. Introduction

Periodontal disease is a chronic, infectious, inflammatory pathology determined by a dysbiotic subgingival biofilm. Its evolution can lead to the destruction of the supporting tissues of the teeth by advanced loss of connective attachment and alveolar bone resorption [1]. The etiopathology of periodontal disease is multifactorial and can be summarized by a sequence of complex links between microorganisms in dental biofilms and the immunoinflammatory response of the host. Dental plaque microorganisms act on periodontal tissues by direct and indirect mechanisms, releasing molecules that directly destroy the tissues and secondarily activate the immune-inflammatory response [2]. Clinical and immunological aspects of periodontal disease are enhanced by systemic factors such as diabetes, smoking, alcohol consumption, and stress, which are directly associated with severe periodontitis [3,4]. At the molecular level, there has been a reported inflammatory response that is associated with active periodontitis and can affect the general homeostasis of the patient. This response can lead to general manifestations far away from the oral cavity [5]. With all the systemic implications that periodontal disease has, it certainly plays a key role in affecting or maintaining overall health, having a major impact on quality of life [3,6]. The therapeutic protocols for treating periodontitis have undergone major changes in the past due to improvements in research on biomaterials and tissue engineering. The best way to evaluate the outcome of the novel protocols before clinical implementation is by using them on animal models. Animal models need to be expanded in the research field prior to clinical trials whenever a new therapy or material is proposed to evaluate the outcome of periodontal disease treatment [7,8].

The induction of periodontal disease by ligature placement in animals is widely used [8,9,10]. A rat model of induced periodontitis appears to be the best option considering time, cost, the results, and number of sacrificed animals for short-term studies [5,7,8]. The experimental model is the best link between the hypothesis and clinical treatment. The ligature-induced model is the most used model for inducing periodontitis. The literature describes methods of inducing periodontal disease by placing a ligature around the rats’ molar teeth (1st or 2nd) [3,6]. Due to the difficulties of reaching the molar area of the rats for further clinical evaluation, different techniques of inducing periodontal disease by placing a ligature around the rat incisors have been proposed [5,11,12]. Undoubtably, there are major advantages to using the incisor technique. The incisor is more commonly used due to the facilitated access and the easygoing operatory technique [13,14,15].

The aim of this study was to evaluate and compare the clinical, biochemical, and histological changes that occur after inducing periodontal disease in the molars vs. the incisors of rats, to establish if the modifications of the aforementioned parameters are the same for the two models and to validate the one we can use as a standard for further studies.

## 2. Materials and Methods

### 2.1. Study Design

The experiment was conducted inside the Laboratory Animal Facility—Centre for Experimental Medicine, Iuliu Hațieganu University of Medicine and Pharmacy. All protocols described below were approved by the Institutional Ethics Committee of the University of Medicine and Pharmacy Iuliu Hațieganu Cluj Napoca (Protocol no. 87/03.03.2017) and The National Sanitary Veterinary and Food Safety Authority (authorization no. 39/10.03.2017). They were performed and conducted in accordance with present laws regarding animal welfare and ethics in animal experiments (Directive 86/609 EEC/1986; Romanian Law 205/2004; Romanian Law 206/2004; Romanian Law 471/2002; Romanian Law 9/2008; Romanian Order 143/400). This study was conducted on 30 adult male Wistar rats obtained from the above mentioned Animal Facility with an initial mean weight of 219.83 g and a standard deviation of 24.91. The animals were selected according to the inclusion criteria (male sex, Wistar race, weight between 200–300 g, 18–20 weeks of age). The exclusion criteria for this study were visible signs of disease and animals that were primarily used in other experimental activities. The animals were kept in plastic type II-L open-top cages (Tecniplast Buguggiate, Italcages, Varese, Italy). They had an acclimatization housing period of 2 weeks before beginning the experiment. Five animals were housed in each cage and maintained under a 12-h light/dark cycle in a temperature- and humidity-controlled room (23 ± 1 °C and 50 ± 5% relative humidity) with access to standard rat chow pellets and water ad libitum. Animals were divided into three groups: two test groups and one control group. All three groups received the same housing and feeding regimen. The control group (CONTROL) included 10 subjects. For the subjects in this group, we performed blood sampling and local anesthesia using the same technique and dosage as for the subjects in the test groups. This group was used to identify the changes in the monitored parameters influenced by the stress induced during the blood sampling or anesthesia procedure or by the substances used for the local anesthesia. The first test group (TEST1) included 10 subjects. For the subjects in this group, we applied the protocol for inducing experimental periodontitis using incisor ligature. The second test group (TEST2) included 10 subjects as well. For the subjects in this group, we applied the protocol for inducing experimental periodontitis using molar ligature.

### 2.2. Blood Sampling

Two blood samples of 1.5–2 mL were obtained from each animal by retro-orbital sinus puncture after general sedation with isoflurane (Aerrane, Baxter, Berkshire, UK) using a standard vaporizer (EZ-Anesthesia^®^; E-Z Systems, Bethlehem, PA, USA). The first blood sample was obtained on the first day of the experiment (Initial), and the second sample was obtained on the 7th day of the experiment (Final). The blood was stored in tubes with ethylenediaminetetraacetic acid (EDTA) as an anticoagulant. The total blood count was completed within 2 h of sampling, and the rest of the blood was centrifuged for 10 min at 3000 rpm. Plasma was separated and kept in Eppendorf tubes at −20 °C until further use in the immunoassays.

### 2.3. Anesthesia Protocol

After blood sampling, general anesthesia was performed by an intraperitoneal injection with a mixture of 10% ketamine and 2% xylazine (2:1), 0.12 mL/100 gr body weight, using a 1 mL syringe with a 26 G—0.45 × 12 milimeters detachable needle (Omnifix^®^-F Solo, BBraun, Melsungen, Hessen, Germany). The correct dosage was calculated according to the weight measurements performed after sedation but before blood sampling.

### 2.4. Ligature-Induced Periodontitis Protocol

**Figure 1:**
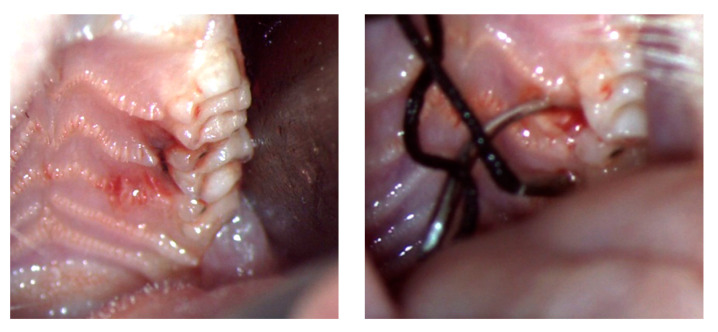
Ligature application around molars.

After anesthesia, the subjects were placed on the surgical table. One member of the team was in charge of keeping the mouth open using a sterile gauze and tissue retractors. A 4.0 silk suture with a 7 mm needle (BBRAUN Silkam^®^, Silk suture 4-0, 7 mm, 45 cm, 3/8 reverse cutting needle, Melsungen, Hessen, Germany) was used. The application of the suture was performed with two microsurgical Castroviejo needle holders using a dental spatula as a tissue retractor for the cheek. All procedures were performed under magnification with 5.0× loupes (Zeiss, Germany) with a 50,000 lux WD 300 mm intensity head light. Incisor—The silk tread was placed around the lower incisors as ligatures in an “8”, using the protocol described in the literature [5,13,16]. Molar—The application of silk was performed around the second maxillary molar on the left side for every subject in the second test group (Test 2). The second molar was used due to the presence of the mesial and distal contact points, which lower the risk of the ligature sliding, Figure 1.

The needle was first inserted from buccal to palatal at the mesial side of the second molar and then from palatal to buccal at the distal side of the tooth. The knot was fixed on the buccal side after the suture was inserted into the sulcus with a dental spatula. The postoperative care was the same as in any surgery with general anesthesia in an experimental animal. To prevent corneal damage, an eye ointment (kanamycin sulfate, Antibiotice SA^®^, Iași, Romania) was used [17]. At the end of the experiment (7 days after placing the ligatures), after blood sampling, the subjects were sacrificed by an overdose of anesthetic, and the samples were prepared for histological handling.

### 2.5. Analytical Methods

#### 2.5.1. Clinical Investigation

Clinically, the evaluation was made at the beginning of the study and at the end of the study. The parameters assessed were body weight, probing depth, gingival bleeding score, and tooth mobility. The probing depth and the bleeding index were evaluated using a periodontal probe (PCP UNC-15, HuFriedy^®^, Chicago, IL, USA). Mobility was assessed with the help of 2 dental instrument handles. All parameters were recorded in each subject’s chart. Tooth mobility was recorded following the scoring system described by Xu and Wei [18]: The mobility of the left second molars and the lower incisors was classified as follows: 0 = physiological mobility; 1 = slight mobility (buccal-palatal); 2 = moderate mobility (buccal and mesial-distal); 3 = severe mobility (tooth moves in and out of the socket). Second molars and lower incisors were probed in the region of the periodontal pocket or gingival sulcus for ten seconds, and the classification of the Gingival Bleeding Index (GBI) as described by Liu, Li, Zhou, Dong and Wen [19] was used, conferring scores from 0 to 5: score 0: Gingival Margin and gingival Papilla (GMP) are healthy; score 1: GMP mildly inflamed, no bleeding; score 2: GMP mildly inflamed, changes in color, absence of edema, punctate hemorrhage; score 3: GMP moderately inflamed, changes in color, mild edema, and bleeding in the gingival crevice; score 4: GMP severely inflamed, changes in color, severe edema, with blood flowing out of the gingival crevice; score 5: GMP severely inflamed, changes in color, severe edema, ulceration, and spontaneous bleeding, with blood observed flowing out of the gingival crevice.

#### 2.5.2. Laboratory Investigation

The following parameters were recorded after the hematological analyses: total leukocytes, neutrophils, lymphocytes, monocytes, eosinophils, and platelets. To quantify the host inflammatory response, interleukin-1 alpha (IL-1α), tumor necrosis factor-alpha (TNF-α), and high-sensitivity C-reactive protein (hsCRP) plasma concentration measurements were performed. Blood was analyzed using a Sysmex XT-1800i automated hematology analyzer (Sysmex Corporation, Kobe, Japan). The IL-1α, TNF-α, and hsCRP analyses were performed using commercial supplies for research: Rat Interleukin 1-α ELISA (RAF047R), Rat TNF-α ELISA (RAF130R), and Rat hsCRP ELISA (RH951CRP01R) (Bio Vendor^®^ Research and Diagnostic products, Brno, Czech Republic) commercialized and distributed in Romania by ORION Biologics (Cluj-Napoca, Romania).

### 2.6. Histological Investigation

The incisors and molars to which the ligature was applied were dissected together with the part of the jaw in which they were fixed by sectioning with a milling cutter. The harvested pieces were fixed in 10% formalin for 7 days, decalcified with trichloroacetic acid for 4 weeks, dehydrated in ethyl alcohol, clarified with 1-butanol and embedded in paraffin. Care was taken to obtain histological sections in which the 2nd molar and the incisor, the alveolar bone crest, and the coronal and root pulp chambers were clearly identified. When these criteria were met, serial sections with a thickness of 6 μm were made, and the most representative sections (the ones who met all the above mentioned criteria) were colored by Goldner’s trichrome stain method. Histological preparations were examined using a confocal microscope (Olympus BX 41, lens 12.5/0.25, Tokyo, Japan) equipped with an Olympus E-330 (Tokyo, Japan) digital imaging camera.

### 2.7. Statistical Investigation

The statistical handling was performed using IBM^®^ SPSS^®^ Statistics 25.0 (IBM Inc., New York, NY, USA) and the Microsoft Excel application. The data are presented as the mean ± standard deviation of the mean (SD). Normal distribution was assessed using the Shapiro–Wilk test, where *p* values > 0.05 suggested that the data came from a normally distributed population. The comparison between the evaluated parameters of the three groups on the two different evaluation intervals was performed with paired Student’s *t* test for paired samples if data were normally distributed or Wilcoxon signed-rank test if data were not normally distributed where *p* values > 0.05 are considered statistically significant.

## 3. Results

### 3.1. Clinical Oral Characteristics

**Figure 2:**
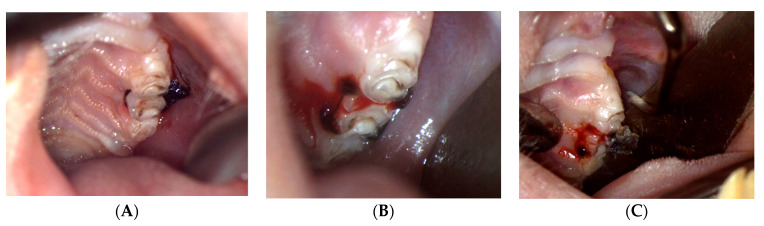
Clinical aspect of the gingiva at the end of the experiment. (**A**)—presence of the ligature at the end of the experiment; (**B**)—bleeding on probing at ligature removal; (**C**)—presence of inflammation, food debris and necrotic tissue.

The clinical signs registered in the examination chart were related to the aspect, contour, and color of the soft tissues surrounding the teeth. On the first day of the experiment, the gingival tissues had a normal appearance, smooth texture, and light pink color. The attached gingiva was firmly enclosed to the underlying structures, whereas the free gingival margin had a sharp contour following the CEJ (cemental-enamel junction) of the adjacent teeth. At the end of the experiment, the clinical aspect of the gingiva was altered. For the molar test group, the macroscopic appearance became cyanotic, with significant edema surrounding the examined teeth. The free gingival margin became irregular presenting food debris (Figure 2).

For the incisor-induced periodontitis model, the gingival modifications were less intense and present only on the lingual side of the incisors. The buccal side of the teeth for this group had a normal aspect with the silk thread situated approximately at 1.5–2 mm above the gingival sulcus. The mean values and standard deviations for clinical parameters (mobility, inflammation, weight) of the three groups from both examination moments are presented in Table 1.

Inflammation occurred in both groups with a statistically significant difference from baseline, but it was more pronounced in Test 2 group. In Test 1 group, the average value of the GBI score tripled at the end of the experiment, whereas the mean value of the tooth mobility score doubled (*p* < 0.01). In Test 2 group, the same average value of the GBI was almost fivefold higher, whereas the mean value of the tooth mobility score tripled (*p* < 0.01). Regarding weight, there were no statistically significant differences in any of the test groups or the control group. However, for the control group, the increase in weight was higher (from 236.63 ± 23.14 to 253.88 ± 22.36) than in the test groups (223.4 ± 22.98 to 231.6 ± 16.93 for Test 1 and 217 ± 29.47 to 211.90 ± 21.06 for Test 2).

### 3.2. Biochemical Characteristics

The mean values and standard deviations for biochemical inflammation markers (hsCRP, TNF-α, and IL1-α) were compared both at the beginning and at the end of the experiment for the three groups included in the study (Table 2).

For the control group, there were no major changes in the evaluated parameters. For the test groups, all parameters increased statistically significantly (*p* < 0.01), except the mean value of IL1-α in the Test 1 group, which had a moderate increase (from 36 ± 15.74 to 51 ± 24.51, *p* = 0.12). The same parameters were compared at the end of the experiment between the two test groups, Test 1 and Test 2 (Table 3).

All the mean values for the evaluated inflammatory markers showed a higher rate for the Test 2 group with a statistically significant difference for hsCRP and TNF-α (*p* < 0.01) and IL1-α (*p* = 0.05). The mean values and standard deviations for biochemical parameters (leukocytes, neutrophils, eosinophils, lymphocytes, monocytes, platelets) were compared both at the beginning and at the end of the experiment for the two test groups (Table 4).

For the control group, the mean values of the evaluated parameters were observed to be steady. For Test 1 group, a statistically significant increase (*p* < 0.05) was noticed for leukocytes and platelets mean values. The eosinophils mean value decreased significantly (*p* < 0.05), whereas the neutrophils, monocytes, and lymphocytes mean values increased slightly (*p* > 0.05). For the Test 2 group, the mean values of eosinophils decreased (*p* = 0.09), monocytes showed an insignificant increase (*p* = 0.71), and all the other investigated hematological parameters increased significantly (*p* < 0.05) from the beginning to the final evaluation.

### 3.3. Histological Characteristics—Descriptive Histology

**Figure 3:**
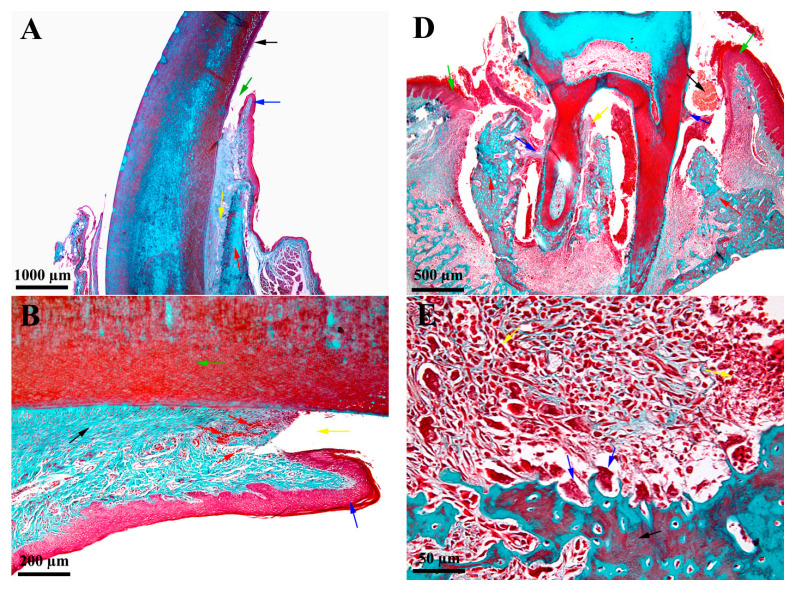

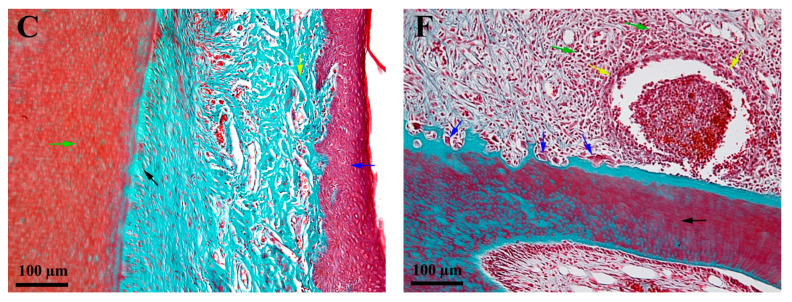
Histological aspects; A-C incisors; D-F molars; Goldner’s trichrome stain (**A**)—black arrow—incisor crown; blue arrow—gingival tissue; green arrow—sulcus; red arrow—alveolar bone; yellow arrow—alveolar ligament; (**B**)—green arrow—dentin; blue arrow—gum; yellow arrow—sulcus; red arrow—discreet congestion of small superficial vessels; black arrow—alveolar ligament; (**C**)—green arrow—dentin; blue arrow—gingival epithelium; yellow arrow—lamina propria; black arrow—alveolar ligament; (**D**)—black arrow—ligature thread; blue arrow—alveolar ligament necrosis; yellow arrow—interradicular necrosis; red arrow—alveolar bone; green arrow—gingiva; (**E**)—black arrow—alveolar bone; blue arrow—osteoclast erosion of the alveolar bone; yellow arrow—inflammatory infiltrate; (**F**)—black arrow—the root of the molar; blue arrow—osteoclast erosion of the root cementum; yellow arrow—incipient granuloma; green arrow—inflammatory infiltrate.

The histologic evaluation was performed one week after the placement of the silk treads for both test groups. The ligatures were present in both study groups, but due to technical difficulties when preparing the probes for histological evaluation for the incisor group (Test 1), the tread was lost during the fixing of the segment. On the incisor pattern, the ligature thread did not cause advanced and extensive periodontitis lesions but only zonal, discrete, and superficial lesions (Figure 3A–C). These discrete lesions are represented by zonal erosion of the structures at the level of the sulcus bottom and moderate congestion of the small blood vessels in the immediate vicinity (Figure 3B). There was no inflammatory infiltrate, and the alveolar ligament, bone and cement were intact. The lesions are discreet with an aspect of mechanical trauma, without triggering inflammatory processes (Figure 3C). In the molar model, the necrotic inflammation led to the disorganization (disappearance) of the alveolar ligament on a very large surface, and the remaining molar was anchored to the alveolar bone only partially on a small surface at the top of the roots (Figure 3D–F). The process was so advanced that it also included soft interradicular structures (Figure 3E). A massive inflammatory infiltrate containing osteoclastic precursors and osteoclasts in large numbers was found in the vicinity of the affected structures. These osteoclasts caused a gradual lysis of the alveolar bone, which appeared much thinner and apically positioned (Figure 3E). The osteoclasts also acted on the roots, where the lesions caused by them were present for the time being only at the root cementum (Figure 3F). Overall, the lesions were so extensive and advanced that the anchorage of the molar in the dental alveola was very weak and its mobility very high.

## 4. Discussion

The present study compared the clinical characteristics, histopathological findings, and systemic inflammatory response of ligature-induced periodontitis in rats for two models (molar and incisor) proposed by the literature to evaluate the best option for further experimental studies. Periodontitis does not naturally affect rats, and the methods of inducing periodontitis in this type of animal model require bacterial inoculation or ligature placements that lead to mechanical irritation, bacterial colonization, gingival inflammation, and eventually bone loss [8,20,21]. While the molar pattern inducing periodontitis is well documented in the literature [8,15,22,23], the incisor model was recently proposed [5,13,16]. The aim of the study was to determine whether the incisor model is reproducible and whether it offers the same results in terms of periodontal disease induction as the molar pattern.

### 4.1. Clinical Oral Aspects

The signs of gingivitis in the rat model resemble the signs of gingivitis in humans: changes in the color, contour, consistency, and bleeding on probing recorded at the end of the experiment are similar to human gingivitis [24]. In humans, if the local inflammation of the gingiva is not treated, it will develop deeper in the tissue structures and will eventually cause attachment loss and bone resorption. Periodontitis appears whenever the attachment apparatus of the teeth is affected by this extended inflammation [25,26,27]. In rat-induced periodontitis, bone resorption and inflammation are suggested to be initiated by mechanical trauma produced by the ligature [22]. The values of the mobility and inflammation parameters increased statistically significantly for both test groups at the end of the experiment. An important aspect to be considered here is that the rat incisor has physiological mobility compared to the molar. Thus, we suggest that the modifications in the molar area were even more complex. Although without statistical significance, a slight decrease in the mean weight from 217 ± 29.47 g to 211.90 ± 24.06 g for the Test 2 group was observed, whereas the control and Test 1 groups increased in weight. This finding could suggest a general homeostasis effect due to periodontal inflammation.

### 4.2. Biochemical Aspects

Regarding the immune inflammatory response, an important outcome of the study is the significant increase in the values of the proinflammatory mediators. C-reactive protein (CRP) is found in blood plasma, and circulating concentrations rise in response to inflammation. It is an acute-phase protein of hepatic origin that increases following interleukin-6 secretion by macrophages and T cells. CRP is synthesized by the liver in response to factors released by macrophages and fat cells (adipocytes) [28,29]. Interleukin 1 alpha (IL-1α) is a cytokine of the interleukin 1 family that in humans is encoded by the IL1A gene. In general, interleukin 1 is responsible for the production of inflammation and the promotion of fever and sepsis. IL-1α is produced mainly by activated macrophages and neutrophils, epithelial cells, and endothelial cells. It possesses metabolic, physiological, and hematopoietic activities and plays one of the central roles in the regulation of immune responses. It binds to the interleukin-1 receptor. This pathway activates tumor necrosis factor-alpha [30,31,32,33]. The primary role of TNF is in the regulation of immune cells. TNF, as an endogenous pyrogen, is able to induce fever, apoptotic cell death, cachexia, and inflammation, inhibit tumorigenesis and viral replication, and respond to sepsis via IL-1 and IL-6-producing cells [34]. All three parameters increased significantly in the two test groups (Test 1 and Test 2) at the end of the experiment compared to baseline, except IL-1 alpha for the Test 1 group, for which the increase was not statistically significant. This finding suggests that the inflammation that occurred in the Test 1 group could be caused by a local trauma and, if left untreated, could be an autolimited infection. Comparing the differences between the two test groups at the end of the experiment, all three parameters were statistically significantly higher for the Test 2 group, which demonstrates a higher proportion of inflammation for molar induced periodontitis. Our findings regarding the immune inflammatory response are similar to other studies in the literature that evaluated the association between this response in periodontitis and systemic diseases (cardiovascular, neurological, metabolic, etc.) [35,36,37,38]. The influence of local inflammation on homeostasis was evaluated by the total count of leukocytes. Neutrophils are the ones that particularly increase in the early stages of inflammation. While the difference between the total count of leukocytes at the beginning and end of the experiment was statistically significant for both test groups, for Test 1 group, the neutrophils did not increase with a statistically significance (*p* = 0.23). The same count of neutrophils for the Test 2 group was statistically significant *(p* = 0.04). These results suggest a higher rate of neutrophil migration into tissue and initiation of the phagocytosis process for the Test 2 group [39].

### 4.3. Histological Aspectss—Descriptive Histology

The histopathological analysis from our research showed significant alveolar bone loss and intense osteoclastic activity 7 days after inducing periodontitis in the test group involving the molar area (TEST 2). The data obtained were in accordance with previous publications that strictly evaluated the molar area [22,26]. In contrast, for Test group 1 (TEST 1), the data contradicted those of recent publications [5,11,12,13,16]. Our research showed that both the gingival epithelium and the dental-alveolar ligament of the rat incisor appeared structurally intact with superficial inflammation of the lingual soft tissue of the incisor due to mechanical irritation of the silk tread. The clinical evaluation revealed that the position of the tread on the buccal side was approximately 1.5–2 mm coronally from the soft tissue margin. This finding is explained by the physiological difference in the position of the soft tissue of the rat incisor (buccal vs. lingual) due to the inclination and the axis of the incisors. The rat incisor is rootless and grows continually. If they do not have material to chew or if they have a malocclusion, the incisors will not wear normally, leading to problems with mouth closure [8,28], which explains the possible migration of the thread.

## 5. Conclusions

The ligature-induced periodontitis model has limitations and will never reproduce all aspects of periodontal disease in humans. However, animal models need to be expanded in the research field prior to clinical trials whenever a new therapy or material is proposed to evaluate the outcome of periodontal disease treatment. Despite the limits of this study (low number of animals per group, no follow-up after ligature removal), the more accurate and similar the pathology induced in animals is, the higher chances for the treatment or clinical observation to replicate in humans will be. For this reason, there should be a consensus on techniques, protocols, and paths for inducing periodontal disease in rats. The clinical implications of this conclusions are important whenever a new technique or bio-material for periodontal disease treatment emerges on the market. Prior to human studies, all the protocols should be applied and validated on animal models. The results will better replicate the ones expected in humans on a model that best replicates the periodontal inflammation found in humans. This is why the selection of technique and animal model for inducing periodontitis is of crucial importance. The findings of this study with the complex association between clinical, biological, and histological aspects of the two experimental models of periodontal pathology induction in rats suggest that a similar periodontal pathology to the one we find in humans is best replicated in rats with the molar induced periodontitis model and this model, which was validated by the literature should be used for further research whenever a rat periodontitis model is implied.

## Figures and Tables

**Table 1:** Clinical parameters, before and after periodontal disease induction.

Group	Variables	Values		*p-*Value
		Initial	Final	
TEST 1	Mobility	1	1.9 ± 0.32	0.0440 × 10^−6^
	Inflammation	0	2.9 ± 0.74	0.0286 × 10^−8^
	Weight (grams)	223.4 ± 22.98	231.6 ± 16.93	0.3756
TEST 2	Mobility	0.00 ± 0.032	3.2 ± 0.42	0.034 × 10^−11^
	Inflammation	0	4.80 ± 0.42	0.0317 × 10^−16^
	Weight (grams)	217 ± 29.47	211.90 ± 24.06	0.5947
CONTROL	Mobility	0	0	-
	Inflammation	0	0	-
	Weight (grams)	236.63 ± 23.14	253.88 ± 22.36	0.17

Data are presented as mean ± standard deviations.

**Table 2:** Biochemical inflammatory markers, before and after periodontal disease induction.

Group	Variables	Values		*p-*Value
		Initial	Final	
CONTROL	hsCRP (pg/mL)	46.7 ± 7.1	47.9 ± 4.58	0.65
	TNF-α (pg/mL)	42.4 ± 6.53	43.3 ± 6.39	0.75
	IL1-α (pg/mL)	47.5 ± 8.61	50.1 ± 3.81	0.39
TEST 1	hsCRP(pg/mL)	46.4 ± 10.81	86.8 ± 9.97	0.0748 × 10^−6^
	TNF-α (pg/mL)	43.6 ± 8.93	64.5 ± 8.19	0.035 × 10^−3^
	IL1-α (pg/mL)	36 ± 15.74	51 ± 24.51	0.12
TEST 2	hsCRP (pg/mL)	52.1 ± 9.55	117.2 ± 15.73	0.015 × 10^−7^
	TNF-α (pg/mL)	54.5 ± 11.38	106.5 ± 16.72	0.019 × 10^−5^
	IL1-α (pg/mL)	38 ± 13.88	65.8 ± 14.34	0.03 × 10^−2^

Data are presented as mean ± standard deviations.

**Table 3:** Comparative analyses of biochemical inflammatory markers, after periodontal disease induction.

Parameter	TEST 1	TEST 2	*p-*Value
hsCRP (pg/mL)	86.8 ± 9.97	117.2 ± 15.73	0.065 × 10^−3^
TNF—α (pg/mL)	64.5 ± 8.19	106.5 ± 16.72	0.012 × 10^−4^
IL1-α (pg/mL)	51 ± 24.51	65.8 ± 14.34	0.05

Data are presented as mean ± standard deviations.

**Table 4:** Hematological parameters, before and after periodontal disease induction.

Groups	Variables	Values		*p-*Value
		Initial	Final	
CONTROL	Leukocytes [10^3^/µL]	10.12 ± 1.85	11.37 ± 1.34	0.098
	Neutrophils (%)	16.4 ± 0.96	16.01 ± 0.94	0.37
	Eosinophils (%)	1.62 ± 0.78	1.61 ± 0.62	0.97
	Lymphocytes (%)	75.23 ± 0.7	75.24 ± 2.35	0.98
	Monocytes (%)	6.49 ± 0.86	6.16 ± 0.98	0.43
	Platelets [10^3^/µL]	1137.1 ± 109.47	1162.6 ± 225.77	0.75
TEST 1	Leukocytes [10^3^/µL]	9.10 ± 3.02	13.63 ± 3.5	0.006
	Neutrophils (%)	18.03 ± 7.27	22.62 ± 9.2	0.23
	Eosinophils (%)	1.78 ± 0.83	1.15 ± 0.3	0.03
	Lymphocytes (%)	70.8 ± 7.69	76.74 ± 7.92	0.1
	Monocytes (%)	9.79 ± 1.94	10.88 ± 1.78	0.2
	Platelets [10^3^/µL]	1141.8 ± 88.73	1255.4 ± 97.65	0.01
TEST 2	Leukocytes [10^3^/µL]	10.27 ± 2.14	13.14 ± 1.12	0.001
	Neutrophils (%)	19.33 ± 3.31	22.13 ± 3.49	0.04
	Eosinophils (%)	2.01 ± 1.2	1.4 ± 0.72	0.09
	Lymphocytes (%)	70.02 ± 3.71	74.91 ± 4.76	0.01
	Monocytes (%)	8.05 ± 1.95	8.38 ± 2.07	0.71
	Platelets [10^3^/µL]	1009.00 ± 182.71	1227.70 ± 167.35	0.01

Data are presented as mean ± standard deviations.

## Data Availability

Data sharing not applicable.
